# Structural and Biochemical Characterization of a Nonbinding SusD-Like Protein Involved in Xylooligosaccharide Utilization by an Uncultured Human Gut *Bacteroides* Strain

**DOI:** 10.1128/msphere.00244-22

**Published:** 2022-08-31

**Authors:** Alexandra S. Tauzin, Zhi Wang, Gianluca Cioci, Xiaoqian Li, Aurore Labourel, Barbara Machado, Guy Lippens, Gabrielle Potocki-Veronese

**Affiliations:** a TBI, CNRS, INRAE, INSAT, Université de Toulouse, Toulouse, France; b ALBA Synchrotron Light Source, Barcelona, Spain; University of Michigan—Ann Arbor

**Keywords:** xylooligosaccharides, SusC/D, *Bacteroidetes*, uncultured gut bacterium

## Abstract

In the human gut microbiota, *Bacteroidetes* break down dietary and endogenous glycosides through highly specific polysaccharide utilization loci (PULs). PULs encode a variety of sensor regulators, binding proteins, transporters, and carbohydrate-active enzymes (CAZymes). Surface glycan-binding proteins (SGBPs) are essential for the efficient capture of the glycosides present on the cell surface, providing *Bacteroidetes* with a competitive advantage in colonizing their habitats. Here, we present the functional and structural characterization of a SusD-like protein encoded by a xylooligosaccharide (XOS) PUL from an uncultured human gut *Bacteroides* strain. This locus is also conserved in Bacteroides vulgatus, thereby providing new mechanistic insights into the role of SGBPs in the metabolism of dietary fiber of importance for gut health. Various *in vitro* analyses, including saturation transfer difference nuclear magnetic resonance (STD-NMR) spectroscopy, revealed that the SusD-like protein cannot bind to the cognate substrate of the XOS PUL, although its presence is essential for the PUL to function. Analysis of the crystal structure of the SusD-like protein reveals an unfolded binding surface and the absence or inappropriate orientation of several key residues compared with other known SusD-like structures. These results highlight the critical role of the SusD-like protein in the transport of oligosaccharides and provide fundamental knowledge about the structure-function of SusC/D-like transporters, revealing that the binding specificity of SusD-like SGBPs does not necessarily reflect the uptake specificity of the transporter.

**IMPORTANCE** The metabolization of dietary fiber is a crucial function for many gut bacteria, especially *Bacteroidetes*, which are particularly well adapted for recognizing, binding, transporting, and degrading glycosides. In this study, we report the functional and structural characterization of a SusD-like protein involved in xylooligosaccharide utilization by an uncultured gut *Bacteroides* strain. We demonstrate that while this protein is structurally similar to many canonical *Bacteroidetes* surface glycan-binding proteins, it cannot bind the substrate taken up by the cognate SusC-like transporter. This lack of binding might be explained by the absence of several key residues known to be involved in oligosaccharide binding and/or the possible necessity of the SusC-like protein to be present to create a cooperative binding site. The term “surface glycan-binding proteins” generally used for SusD-like proteins is thus not generic. Overall, this study allowed us to revisit the concept of glycoside utilization by *Bacteroidetes*, in particular those strains that feed on the short fibers naturally present in some dietary compounds or on the leftovers of other microbes.

## INTRODUCTION

Humans rely on their microbiota to break down dietary fiber, which is composed of oligosaccharides and polysaccharides that cannot be digested by host carbohydrate-active enzymes (CAZymes) (1). To achieve this, *Bacteroidetes* harbor polysaccharide utilization loci (PULs) that encode proteins involved in sensing, binding, transporting, and degrading the target glycosides into monosaccharides (2, 3). According to the starch utilization system (Sus) paradigm (4–6), polysaccharides are first degraded by enzymes attached to the cell surface. Next, SusD-like substrate-binding lipoproteins, sometimes together with the other surface glycan-binding proteins (SGBPs), SusE and/or -F, selectively recognize and capture the oligosaccharides that will be transported into the cell through the SusC-like protein.

An in-depth characterization of the first starch utilization locus of Bacteroides thetaiotaomicron to be described showed that the SusD protein has a critical role in addition to its binding ability (6). The deletion of SusD actually resulted in a loss of growth, suggesting that the presence of SusD is a prerequisite for efficient transport (7–10). SusD and SusC interact to form a complex (11). Recent findings concerning the structures of SusC/D-like complexes provided new insights into the molecular determinants of the protein-protein and glycan-protein interactions involved (12, 13). The SusC/D-like pair is well conserved among *Bacteroidetes*, highlighting its crucial role in glycan uptake, and the presence of genes encoding SusD-like proteins is crucial for predicting PULs (14).

Dozens of *Bacteroidetes* strains have been shown to use xylan as their main source of carbon (15–25). Xylan is a major constituent of hemicelluloses, an abundant component in plant cell walls, particularly in cereals, fruits, and vegetables (26). The conserved backbone of xylan is composed of a linear β-(1-4)-d-xylopyranosyl chain, which may be decorated with arabinofuranosyl, glucopyranosyl, or uronic acid derivatives (26). Xylooligosaccharides (XOSs) are hydrolyzed from xylan or are found naturally in fruits, vegetable bamboo, and honey (27). XOSs have a range of health benefits, even at a lower dose than fructooligosaccharides, and are thus considered prebiotic candidates (27–30). To date, only two PULs specific for xylan utilization by Bacteroides ovatus ATCC 8483 (PUL-XylS and PUL-XylL) have been fully biochemically characterized. Functionally, they show different specificities depending on the structural complexity of the xylan (21). In addition, in 2016, we identified an XOS-specific PUL from an uncultured gut *Bacteroides* strain and characterized its protein components through recombinant expression in Escherichia coli (31, 32). This locus encodes a SusC/D-like pair and a major facilitator superfamily (MFS) transport system capable of XOS uptake up to a degree of polymerization of 3 (DP3) and DP4, respectively. We also demonstrated that the deletion of the SusD-like protein abolished the growth ability of the recombinant E. coli cells on XOS, consistent with the results obtained with other SusD-like proteins. Furthermore, this PUL shares 99% DNA sequence identity with a Bacteroides vulgatus ATCC 8482 PUL (BVU_0037 to BVU_0043). We previously proved that this PUL is involved in linear XOS utilization by the Bacteroides vulgatus ATCC 8482 strain (32). On XOS, its growth is very robust, while on arabinoxylooligosaccharides (AXOSs), it is delayed and takes place at a lower rate. In addition, this strain is unable to utilize complex heavily decorated xylans or arabinoxylan, although rarely, it may utilize simpler xylans from beechwood and birchwood (32).

In the present study, we investigated how the SusD-like protein from this XOS-targeting PUL contributes to the utilization of its cognate substrate. By using various *in vitro* techniques to investigate the binding ability of this SusD-like protein and analyzing its crystallographic structure, we provide new mechanistic insights into XOS uptake by *Bacteroidetes*.

## RESULTS

### F5_SusD-like cannot bind to XOS. (i) Determination of the binding ability by AGE, ITC, and DSF.

The XOS locus encodes a SusD-like protein (referred to as F5_SusD-like here) sharing 99.4% identity with the SusD-like protein (BVU_0037) from Bacteroides vulgatus ATCC 8482 (with four differences at positions N249, D444, A446, and A516 corresponding to D, G, P, and V residues in BVU_0037, respectively).

**FIG 1:**
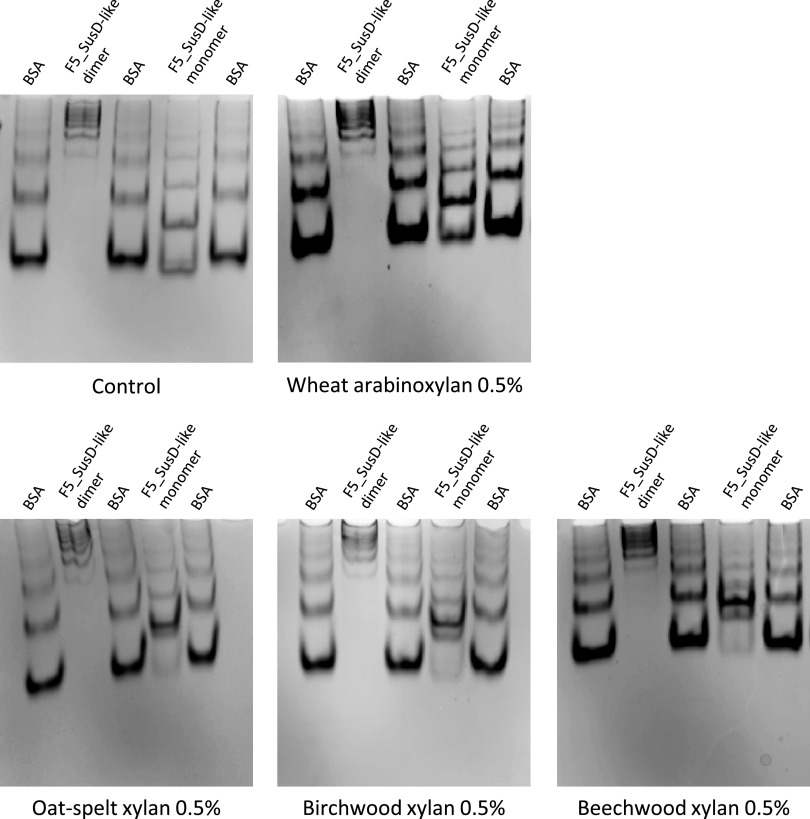
Affinity gel electrophoresis of F5_SusD-like on a gel containing no polysaccharide (negative control) and on gels with 0.5% (wt/vol) different xylans.

The recombinant protein was expressed in E. coli without the predicted signal peptide and N-terminal lipidation site. The ability of F5_SusD-like to bind several soluble glycans, including xylans and arabinoxylan, was assessed by affinity gel electrophoresis (AGE) (Fig. 1; see also Fig. S1 in the supplemental material). As shown by size exclusion chromatography (Fig. S2), F5_SusD-like forms monomers and dimers in solution. The dimer showed no difference in migration in the presence or absence of glycan. In contrast, monomer migration was slightly delayed by 27, 28, and 31% in the gels containing beechwood, birchwood, and oat spelt xylans, respectively. No migration delay was observed with wheat arabinoxylan. The observed smears suggest that the binding of the monomer to xylans could be unstable due to a weak affinity. F5_SusD-like is retained in the gel containing xylans to a much smaller degree than what was observed for all other characterized SusD-like proteins that efficiently bind polysaccharides (9, 10). These results indicate a weak affinity of F5_SusD-like for xylan, impaired by the presence of side chains on the xylan backbone. Isothermal titration calorimetry (ITC) was attempted using XOS as a ligand, but no binding could be detected (data not shown). Finally, differential scanning fluorimetry (DSF) was performed, but no significant change in the intrinsic fluorescence of F5_SusD-like was observed in the presence of birchwood, beechwood, or oat spelt xylans or barley β-glucan, carboxymethylcellulose, laminarin, wheat arabinoxylan, or xyloglucan (data not shown). These results indicate that the thermal stability of F5_SusD-like was not affected by significant ligand binding, contrary to what could be observed using DSF for genuine polysaccharide-binding proteins (33).

10.1128/msphere.00244-22.1 FIG S1 Affinity gel electrophoresis of F5_SusD-like on a gel containing no polysaccharide (negative control) and on gels with 0.5% (wt/vol) different β-glucans. Download FIG S1, TIF file, 0.6 MB. Copyright © 2022 Tauzin et al. 2022 Tauzin et al. https://creativecommons.org/licenses/by/4.0/ This content is distributed under the terms of the Creative Commons Attribution 4.0 International license.

10.1128/msphere.00244-22.2 FIG S2 Analysis of F5_SusD-like oligomerization. (A) Elution profile of the protein when injected onto a Superdex 200 16/600 column in buffer containing 20 mM Tris-HCl and 150 mM NaCl (pH 7.5). (B) SDS-PAGE analysis of the fractions collected for panel A. Download FIG S2, TIF file, 0.4 MB. Copyright © 2022 Tauzin et al. 2022 Tauzin et al. https://creativecommons.org/licenses/by/4.0/ This content is distributed under the terms of the Creative Commons Attribution 4.0 International license.

### (ii) Determination of the binding ability by NMR spectroscopy.

**FIG 2:**
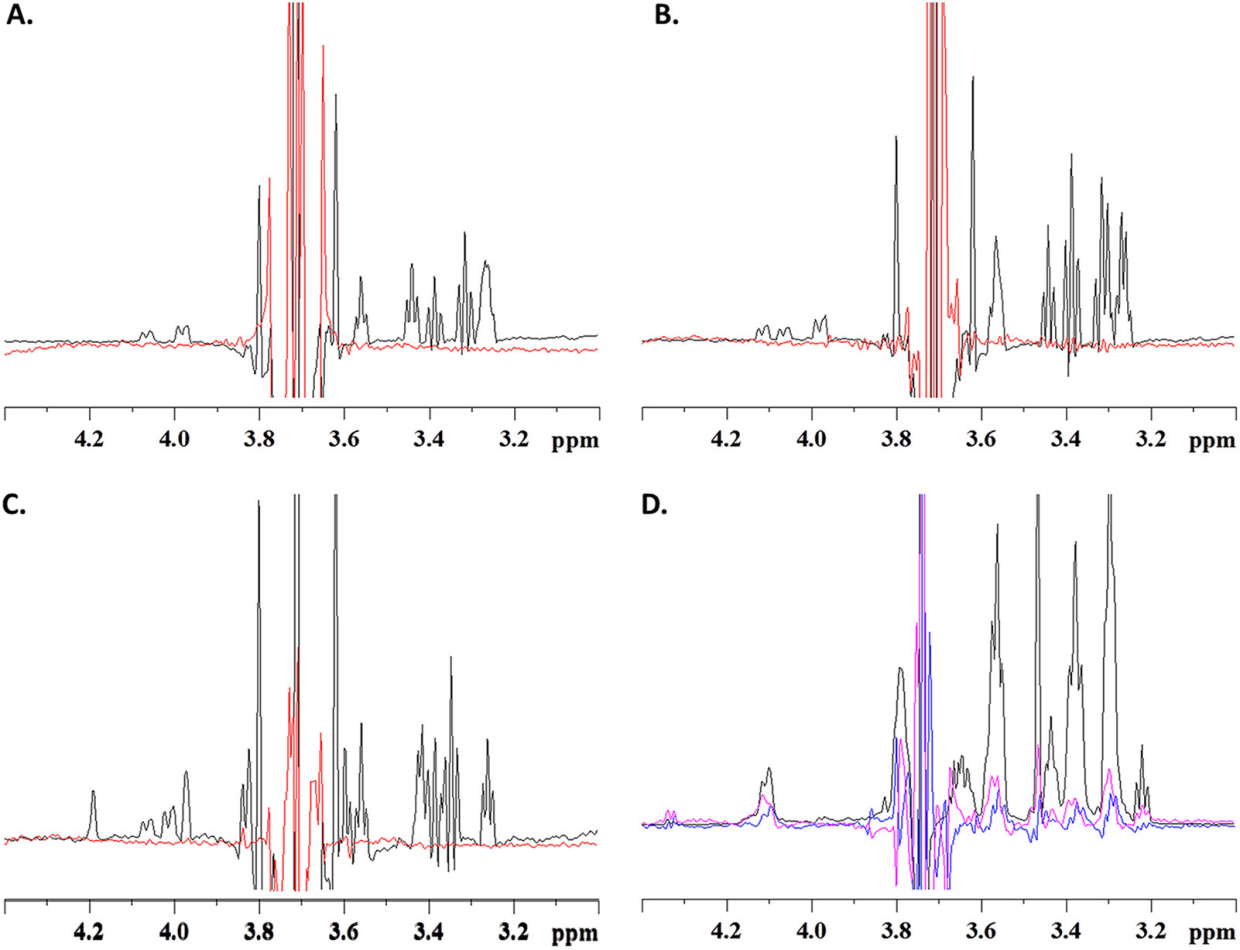
Characterization of the ligand-binding abilities of F5_SusD-like using STD-NMR spectroscopy. (A) Zoomed-in view of the 1D xylobiose proton spectrum centered on the signals at 3.7 ppm (black) and the STD signal of the ligand upon saturation of the protein signals at 0 ppm (red). (B) Same as panel A but for the xylotriose. (C) Same as panel A but for the branched arabinoxylooligosaccharides (AX2 [3^2^-α-l-arabinofuranosyl-xylobiose]). (D) Zoomed-in view of the 1D xylan spectrum (black), the STD signal upon saturation at 10.1 ppm of the isolated xylan (blue), or the xylan in the presence of F5_SusD-like (pink). The increased STD effect in the presence of F5_SusD-like indicates a molecular interaction.

The value of saturation transfer difference nuclear magnetic resonance (STD-NMR) spectroscopy for the direct analysis of protein-ligand interactions with even weak affinities has been shown previously (34). However, to our knowledge, this approach has been used only rarely to analyze the binding ability of carbohydrate-binding proteins associated with CAZymes or with carbohydrate transporters (35, 36). Here, we used this method in order to further characterize the (in)ability of F5_SusD-like to bind to XOS. We first confirmed by one-dimensional (1D) NMR that the F5_SusD-like protein was correctly folded in solution (Fig. S3). The results of the STD experiments with xylobiose, xylotriose, or 3^2^-α-l-arabinofuranosyl-xylobiose (AX2) on the F5_SusD-like protein were negative (Fig. 2A to C), thereby confirming that small xylooligosaccharides do not bind to the protein. A beechwood xylan sample was then prepared, but its opacity indicated that even at 500 μM, the xylan chains assemble into macroscopic structures. The NMR experiment in the presence of the F5_SusD-like protein gave a robust STD signal, but this was also the case when we probed the same xylan sample in the absence of the protein (Fig. S4). Hence, we were unable to distinguish whether binding occurred between xylan and the transporter or between the macroscopic xylan superstructures. To reduce signal saturation due to these macroscopic xylan structures, we saturated the protein indole protons (at 10.1 ppm), where xylan signals could be expected to be reduced. The STD effect with xylan alone was indeed reduced (Fig. S5) and was not only more pronounced in the presence of F5_SusD-like but also increased when we added more protein (Fig. 2D). We thus concluded that F5_SusD-like does not bind short xylooligosaccharides but binds weakly to xylan.

10.1128/msphere.00244-22.3 FIG S3 1D proton spectrum at 298 K of 500 μL of the F5_SusD-like protein at 3.8 mg/mL (54 μM) in Tris buffer. Download FIG S3, TIFF file, 0.01 MB. Copyright © 2022 Tauzin et al. 2022 Tauzin et al. https://creativecommons.org/licenses/by/4.0/ This content is distributed under the terms of the Creative Commons Attribution 4.0 International license.

10.1128/msphere.00244-22.4 FIG S4 (Left) 1D proton spectrum at 298 K of 500 μL of xylan at 500 μM with F5_SusD-like at 0 μM (blue), 15 μM (red), or 27 μM (magenta) in Tris buffer. (Right) STD spectra of the different samples with saturation at 0 ppm for the isolated xylan (blue) in the presence of 15 μM (red) or 27 μM (magenta) F5_SusD-like. The reference xylan spectrum is shown in black. Download FIG S4, TIF file, 0.2 MB. Copyright © 2022 Tauzin et al. 2022 Tauzin et al. https://creativecommons.org/licenses/by/4.0/ This content is distributed under the terms of the Creative Commons Attribution 4.0 International license.

10.1128/msphere.00244-22.5 FIG S5 (Left) 1D proton spectrum of 500 μL of xylan at 500 μM with F5_SusD-like at 0 μM (blue), 15 μM (red), or 27 μM (magenta) in Tris buffer at 298 K. (Right) STD spectra of the different samples with saturation at 10.1 ppm for the isolated xylan (blue) in the presence of 15 μM (red) or 27 μM (magenta) F5_SusD-like. The reference xylan spectrum is shown in black. Download FIG S5, TIF file, 0.2 MB. Copyright © 2022 Tauzin et al. 2022 Tauzin et al. https://creativecommons.org/licenses/by/4.0/ This content is distributed under the terms of the Creative Commons Attribution 4.0 International license.

### Crystallographic structure of F5_SusD-like.

**FIG 3:**
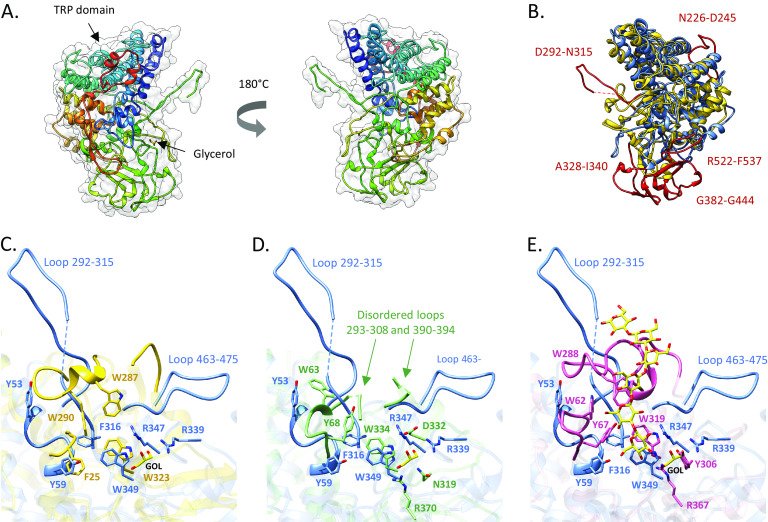
F5_SusD-like crystal structure. (A) Overall structure of F5_SusD-like. (B) Secondary-structure-matching (SSM) superimposition of F5_SusD-like (blue) with GM_SusD (PDB accession number 6GCZ) (the closest structural homolog) (yellow) shown to bind to branched laminarin and linear pustulan. The loops specific for F5_SusD-like are shown in red. (C) Zoomed-in view of the superimposition of F5_SusD-like with GM_SusD. (D) Superimposition of F5_SusD-like with the nonbinding SusD-like protein *Bu*SGBP-A (PDB accession number 7KV1) (green) present in a PUL-degrading β(1,3)-glucan and a mixed-linkage β-glucan. (E) Superimposition of F5_SusD-like with *Bt*SGBP-A (PDB accession number 7KV3) (pink) in complex with laminarihexaose. The glycerol (GOL) present in panels C to E belongs to the F5_SusD-like structure. The figures were created with Chimera (60).

In order to better understand why F5_SusD-like does not bind the XOS substrate targeted by the PUL, we solved its three-dimensional structure by X-ray crystallography. The most similar sequence listed in the Protein Data Bank (PDB) (https://www.rcsb.org/) (37) is that of a SusD-like protein from Parabacteroides distasonis ATCC 8503 (PDB accession number 3OTN). The identity is limited, however, with only 34% identity on a short stretch of the sequence (128 amino acids), which made the structure solution by MR (molecular replacement) a challenging task. We therefore solved it using the MR-SAD (single-wavelength anomalous diffraction) technique (38) and obtained a refined structure at a 2.6-Å resolution. Data collection and refinement statistics are shown in Table 1. Despite the very low sequence similarity with structurally characterized SusD-like proteins, the F5_SusD-like protein displays a canonical “RagB/SusD” fold, which includes the conserved tetratricopeptide repeat (TPR) units. Overall, the structure of F5_SusD-like is similar to those of the 24 other SusD-like proteins that have been listed in the PDB since 2008, when the crystallographic structure of SusD was solved by Koropatkin et al. (7). F5_SusD-like presents a convex surface created by the α-helical bundle and a flat surface on the other side, where the putative sugar binding platform should be located. According to the PDB, no xylan-binding SusD-like protein has been structurally characterized so far. The closest structural homolog identified using the Dali server (http://ekhidna2.biocenter.helsinki.fi/dali/) (39) is the laminarin-binding protein GM_SusD from *Gramella* sp. strain MAR_2010_102 (PDB accession number 6GCZ) (40), with a root mean square deviation (RMSD) of about 2.9 Å. The superimposition of both structures highlights the presence of a large region (A328 to I340 and G382 to G444) specific for F5_SusD-like, which is composed of random coils and some β-sheets (Fig. 3). Another interesting feature is the presence of two loops (e.g., N226 to D245 and R522 to F537) that are inserted between the conserved secondary structural elements. Furthermore, the loop from D292 to N315, which protrudes away from the folded domain, is likely to be disordered in solution, although it is partially stabilized by interactions with the neighboring SusD-like molecules in the crystal (Fig. S6). Comparison with other structurally characterized members of the SusD family shows that these features make F5_SusD-like slightly larger albeit in the same size range as those of other SusD-like proteins from xylan-targeting PULs (Table S1).

**TABLE 1:** Data collection and refinement statistics for the crystal structure of the F5_SusD-like protein^*a*^

Parameter	Value	
	Native	S-SAD
Data collection statistics		
Wavelength (Å)	0.979	2.066
Space group	I4_1_22	I4_1_22
No. of molecules per asymmetric unit	1	1
Cell constants *a*, *b*, *c* (Å)	179.09, 179.09, 182.23	179.84, 179.84, 183.10
Resolution (Å) (range)	50.00–2.65	50.00–3.30
No. of measured reflections	307,172	2,114,678
No. of unique reflections	43,113	22,897
Data completeness (%)	100.0	100.0
Multiplicity (%)	7.1	92.4
*R*_merge_	0.062 (0.736)	0.10 (0.41)
〈*I*/σ(*I*)〉	8.4 (1.0)	5.2 (1.5)
CC_1/2_	0.99 (0.86)	1.00 (0.99)
Wilson B-factor (Å^2^)	65.3	73.7
Refinement statistics		
*R*_work_/*R*_free_	0.185/0.212	
RMSD bond length (Å)	0.070	
RMSD bond angle (°)	1.113	
Ramachandran plot favored/allowed/ outlier regions (%)	97/2/0	
No. of atoms		
Protein	4,849	
Water	16	
Other	6	
Avg B-factor (Å^2^)	87.3	
Cruickshank’s DPI	0.193	
MolProbity clashscore (percentile)	4 (100th)	

a Values in parentheses are for the highest-resolution shell. DPI, diffraction precision index; S-SAD, sulfur single-wavelength anomalous diffraction phasing; CC1/2, Pearson’s correlation coefficient.

10.1128/msphere.00244-22.6 FIG S6 Close-up view of the interaction between the loop spanning positions 292 to 315 and a symmetry-related molecule. Tyr307 is bound in a small amphipathic cavity and stabilized by hydrogen bonds with Asp30, Arg26, and Gln24. The 2*F*_o_-*F*_c_ electron density map (contoured at 1.0 σ) around Tyr307 is shown. Download FIG S6, TIF file, 0.3 MB. Copyright © 2022 Tauzin et al. 2022 Tauzin et al. https://creativecommons.org/licenses/by/4.0/ This content is distributed under the terms of the Creative Commons Attribution 4.0 International license.

10.1128/msphere.00244-22.9 TABLE S1 Molecular weight comparison of F5_SusD-like with SusD-like proteins from xylan-targeting PULs. *Experimentally shown to bind xylan. Bacova_04392 binds to relatively simple xylans, xylohexaose, but not shorter xylooligosaccharides. Bacova_03427 binds to highly decorated corn glucuronoarabinoxylan but not linear xylooligosaccharides. (1) G. V. Pereira, A. M. Abdel-Hamid, S. Dutta, C. N. D’Alessandro-Gabazza, et al., Nat Commun 12:459, 2021, https://doi.org/10.1038/s41467-020-20737-5. (2) A. Rogowski, J. A. Briggs, J. C. Mortimer, T. Tryfona, et al., Nat Commun 6:7481, 2015, https://doi.org/10.1038/ncomms8481. (3) J. Despres, E. Forano, P. Lepercq, S. Comtet-Marre, et al., BMC Genomics 17:326, 2016, https://doi.org/10.1186/s12864-016-2680-8. Download Table S1, DOCX file, 0.01 MB. Copyright © 2022 Tauzin et al. 2022 Tauzin et al. https://creativecommons.org/licenses/by/4.0/ This content is distributed under the terms of the Creative Commons Attribution 4.0 International license.

We attempted to cocrystallize and soak F5_SusD-like crystals with XOSs ranging from DP2 to DP4, but no complex structure was obtained, confirming the inability of F5_SusD-like to bind to short-chain XOSs. Nevertheless, a glycerol molecule under crystallization conditions is found on the flat surface where the canonical binding site is located. The glycerol molecule is coordinated by two arginine residues (R339 and R347), and W349 makes a hydrophobic stacking platform (Fig. 3). Comparison with GM_SusD shows that two of the three tryptophan residues (W287 and W290) involved in substrate binding are missing in F5_SusD-like (Fig. 3C and Fig. S7A). Comparison to the Bacova_02651 SusD-like protein from Bacteroides ovatus (PDB accession number 5E76) (10), which displays a high glycan-binding ability toward xyloglucan (Fig. S7B), shows that an essential aromatic residue involved in substrate binding (W283) is missing in F5_SusD-like. Moreover, W75, which corresponds to W82 in Bacova_02651, displays a radically different side chain orientation.

10.1128/msphere.00244-22.7 FIG S7 Comparison of the oligosaccharide-binding areas of SusD-like proteins with the homologous region of F5_SusD-like. In each panel, F5_SusD-like is in blue. (A) Comparison of F5_SusD-like with the laminarin-binding GM_SusD protein (PDB accession number 6GCZ) (yellow) from *Gramella* sp. MAR_2010_102. (B) Comparison of F5_SusD-like with Bacova_02651 (PDB accession number 5E76) (cyan) bound to xyloglucan. The stacking platforms identified in GM_SusD and Bacova_02651 are shown with the corresponding residues in F5_SusD-like, which are noneffective in binding XOS. The glycerol (GOL) present in panels A and B belongs to the F5_SusD-like structure. The figures were created with Chimera. Download FIG S7, TIF file, 0.8 MB. Copyright © 2022 Tauzin et al. 2022 Tauzin et al. https://creativecommons.org/licenses/by/4.0/ This content is distributed under the terms of the Creative Commons Attribution 4.0 International license.

Attempting to identify the molecular determinants that make F5_SusD-like a non-glycan-binding protein is particularly difficult, especially since no xylan-binding SusD-like protein is available in the PDB. Nevertheless, a striking feature of F5_SusD-like is the long partially disordered loop (D292 to N315) located close to the canonical binding site. It is noteworthy that some of the tryptophan residues involved in substrate binding in the laminarin-binding SusD-like protein GM_SusD from *Gramella* sp. MAR_2010_102 (PDB accession number 6GCZ) (substrate-binding residues W287 and W290) (40) and in the xyloglucan-binding SGBP-A protein from Bacteroides ovatus (PDB accession number 5E76) (substrate-binding residue W283) (10), which are missing in F5_SusD-like, are carried by a folded loop located precisely in the area from D292 to N315. The presence of a disordered loop close to the binding site is reminiscent of the structure of the SGBP-A protein from Bacteroides uniformis (*Bu*SGBP-A) (PDB accession number 7KV1) (RMSD, 3 Å) (41) (Fig. 3D). This SusD-like protein does not bind to β(1,3)-glucan, while it displays the key binding residues present in the SGBP-A protein from Bacteroides thetaiotaomicron (*Bt*SGBP-A) (PDB accession number 7KV3) (41), which were shown to be involved in substrate binding (Fig. 3E). Tamura et al. correlated this lack of β(1,3)-glucan binding in *Bu*SGBP-A with structural disorder (41). Indeed, three loops critical for shaping the binding site are structurally disordered or deformed in *Bu*SGBP-A (positions 60 to 70, 293 to 308, and 390 to 394, corresponding to positions 54 to 58, 292 to 315, and 463 to 475 in F5_SusD-like).

Finally, the four residues N249, D444, A446, and A516 of F5_SusD-like, corresponding to D, G, P, and V residues in the nearly identical SusD-like protein BVU_0037 from Bacteroides vulgatus ATCC 8482, respectively, are not located in the binding-site area. The oligosaccharide-binding regions of canonical SusD-like proteins are thus strictly identical in F5_SusD-like and BVU_0037, indicating that BVU_0037, like F5_SusD-like, probably does not bind XOSs.

## DISCUSSION

Despite the low sequence identity among SusD-like proteins, the SusC/D-like pair is used as a reference for PUL identification in genomes (14, 42). Indeed, substrate recognition and capture are key steps for glycoside utilization and for bacteria to colonize their habitat. So far, most of the SusD-like proteins that have been biochemically characterized are able to bind the polysaccharide targeted by the PUL but also, to some extent, oligosaccharides resulting from polysaccharide hydrolysis by cell-exposed enzymes. Only in rare cases have SusD-like proteins been shown to be unable to bind glycans, but their function has been investigated using only AGE or ITC (41, 43, 44). Here, we fully characterized a SusD homolog (F5_SusD-like) from an XOS PUL isolated from an uncultured bacterium, which shares 99.4% sequence identity with the B. vulgatus SusD-like protein BVU_0037. Previously, we demonstrated that the PUL containing BVU_0037 was involved in the ability of B. vulgatus to grow on XOS and, to a lesser extent, on branched arabinoxylooligosaccharides, while it utilizes xylans poorly (32). In the present study, we investigated the binding ability of the F5_SusD-like protein. Surprisingly, F5_SusD-like was not able to bind XOSs or AXOSs, while it exhibited a very low affinity for xylan. In contrast, the glycan-binding SusD-like proteins characterized to date display a strong binding affinity in the millimolar-to-micromolar range, depending on the length of the tested substrate. In most cases, the values obtained for complex polysaccharides are in the same range as the values obtained with the oligosaccharides resulting from polysaccharide hydrolysis (10, 45). Rogowski et al. characterized two SusD-like proteins involved in two distinct xylan PULs from B. ovatus (Bacova_04392 and Bacova_03427) (21). In contrast to the F5_SusD-like protein, which belongs to a PUL specific for XOS, these SusD-like proteins showed strong abilities to bind xylan, measurable by ITC, but only Bacova_04392 binds xylooligosaccharides no smaller than DP6 (*K_D_* [equilibrium dissociation constant] = 8.0 × 10^2^ M^−1^ for xylohexaose) (21).

Several studies previously showed that SusD-like proteins are essential for substrate uptake via the SusC-like transporter (7, 9, 10). This is also the case for the F5_SusC/D-like system since when the F5_SusD-like gene is deleted, the F5min_SusΔSusD strain can no longer grow on XOS (32). The F5_SusC/D-like pair is thus able to transport XOS, although the F5_SusD-like protein does not bind them. If the presence of the SusD-like proteins is crucial for glycoside transport, in *Bacteroidetes*, their binding ability is not always required, for the reasons explained here. Both the presence and binding ability of the SusD homolog from the *B. ovatus* transport system are essential for growth on mixed-linkage β-glucans (9). In contrast, genetic complementation using nonbinding SusD-like mutants is sufficient to restore the functionality of the starch transport system in B. thetaiotaomicron and the xyloglucan transport system in *B. ovatus* (7, 10).

As explained previously by Foley and coworkers regarding the Sus (46) and recently by Gray et al. regarding the fructooligosaccharide transport system in B. thetaiotaomicron (12), it might be because in *Bacteroidetes*, the cell surface glycan-binding proteins functionally complement each other in the capture of the polysaccharide. Indeed, Foley et al. (46) showed that when the SusD, -E, and -F proteins of the B. thetaiotaomicron Sus were mutated to nonbinding alleles, growth still occurred on maltooligosaccharides, even in a SusG deletion background. In that study, those authors demonstrated that SusE, independent of its starch-binding sites, can restore glycan uptake by the SusC/D system when the SusD starch-binding ability is abolished. In this context, the most probable hypothesis is that SusE interacts with the SusC/D complex, allowing SusD to open for import. Besides, in the rare examples of nonbinding SusD-like proteins that have been identified so far from loci involved in pectin and β-1,3-glucan utilization, there are additional SGBP- or SusD-like protein-encoding genes in the same locus, suggesting that they compensated the system for glycan binding (43, 44). However, two other nonbinding SusD-like proteins (BT2625 and BT3855) have been identified in the α-mannan utilization loci MAN-PUL1 and MAN-PUL3 of B. thetaiotaomicron, which do not contain any other SusD-like protein or SGBP (47). One cannot exclude that mannan-binding outer surface proteins encoded by MAN-PUL2, which is coexpressed with MAN-PUL1 and MAN-PUL3 during α-mannan utilization, could compensate for the nonbinding SusD-like proteins BT2625 and BT3855 to capture the targeted polysaccharide. Nevertheless, regarding SusD-like proteins that naturally do not bind glycans as isolated proteins, another hypothesis is that their interaction with glycans requires the presence of the cognate SusC-like protein. This hypothesis is in accordance with the data reported previously by Tauzin et al. (10), who showed by reverse genetic analysis that the presence of the SusD-like SGBP-A protein is more critical than its carbohydrate-binding ability for growth on xyloglucan, while the SGBP-B protein is not essential, although it supports the efficient capture of xyloglucooligosaccharides. In the present paper, we confirm that a SusC/D-like transport system should be functional for the uptake of the substrate targeted by the PUL without any binding ability of SusD-like or any other SGBP, supporting a model of glycan import whereby at least the SusC-like and SusD-like proteins must be associated to support glycan uptake.

In order to better understand the molecular determinants of the very weak binding ability of F5_SusD-like, we determined its crystal structure. Although F5_SusD-like could not be cocrystallized or soaked with XOS, a glycerol molecule was found at the canonical oligosaccharide-binding site, bound by R339, R347, and W349, which makes a hydrophobic stacking platform. This stacking platform might be involved in the very weak binding of xylans by F5_SusD-like. However, by comparing it with solved SusD-like structures, we hypothesized that the lack of genuine binding affinity could be due to some missing aromatic residues as well as an unfolded binding surface with the loop spanning D292 to N315 being partially disordered. Also, SusD-like affinity is driven by the surface complementation between the protein and the substrate rather than by the affinity of individual chemical groups for the substrate, as proposed in the very first description of the structure of SusD-like (7) and confirmed subsequently (9, 13, 40, 48–50). Thus, the role of “nonbinding” F5_SusD-like appears to be to effectively complement the SusC-like transporter and activate its functionality. This was first proposed by Glenwright et al. (13), who solved the crystal structure of a quaternary complex from B. thetaiotaomicron BT2261–BT2264, including the SusD-like (BT2263) and SusC-like (BT2264) transporters. Using a variety of techniques, those authors proposed a “pedal bin” mechanism in which SusD-like moves away from SusC-like in a hinge-like fashion in the absence of a ligand to expose the substrate-binding site to the extracellular milieu. According to this model, which was further confirmed by Gray et al. (12), SusC-like performs its function only when in complex with SusD-like, which acts as a lid that can open and close the transporter. Because SusC-like and SusD-like are associated proteins *in vivo*, the most probable hypothesis for SusD-like proteins that are not able to bind glycans as isolated proteins is that binding requires the presence of the cognate SusC-like protein. Indeed, the structural analysis of SusD-like BT1762 alone and in a complex with SusC-like BT1763 shows that a disordered loop located in the same area of positions 292 to 315 in F5_SusD-like is stabilized in the complex by the long hinge 2 loop of BT1763 (see Fig. S8 in the supplemental material). The hinge 2 loop is likely to be important for lid opening and is responsible, with hinge 1, for the majority of the interactions between BT1762 and BT1763 in the open state (12). Moreover, hinge 2 carries a phenylalanine residue (F649) making a hydrophobic interaction with the substrate. The proximity of this connection area to the SusD-like canonical binding site suggests that glycan recognition requires the presence of the cognate SusC-like protein. This could also be a hypothesis to explain why a single substitution that abolishes levan binding to BT1762 (W85A) *in vitro* leads to no growth defect *in vivo*. As discussed by Gray et al. (12), the context of the intact transporter ensures that the effects of SusD-like point mutations are much less dramatic *in vivo*. Among the amino acids composing the partially disordered loop at positions 292 to 315 in F5_SusD-like are 1 histidine, 3 tyrosine, and 2 glutamate residues. It would be interesting to know whether these residues are involved in the interaction with F5_SusC-like and/or whether the loop is folded in a way that enables some of these residues to form an extended binding site. Obtaining the three-dimensional (3D) structure of F5_SusC/D-like in complex with XOS would allow us to answer this question.

10.1128/msphere.00244-22.8 FIG S8 Structural comparison of SusD-like proteins alone and interacting with cognate SusC-like proteins. (A) Comparison of F5_SusD-like (blue) with BT1762 crystallized without BT1763 (PDB accession number 6GCZ) (salmon). (B) The structure of the BT1762-BT1763 complex (PDB accession number 6Z9A) (salmon, SusD-like; green, SusC-like) shows the molecular determinants for fructooligosaccharide binding. The figures were created with Chimera. Download FIG S8, TIF file, 1.3 MB. Copyright © 2022 Tauzin et al. 2022 Tauzin et al. https://creativecommons.org/licenses/by/4.0/ This content is distributed under the terms of the Creative Commons Attribution 4.0 International license.

Finally, nonbinding or very weakly binding SusD-like proteins can be found in PULs, including (i) a SusC/D-like pair and an additional SGBP (43), (ii) two SusC/D-like pairs and an SGBP (44), or (iii) only one SusC/D-like pair (47; this study). The presence of an additional SGBP or a SusC/D-like pair in the same locus as that of a nonbinding SusD-like protein suggests compensation for the binding function lacking in the related SusD-like protein. In contrast, as previously hypothesized (47), the PULs containing only a SusC/D-like pair for which SusD-like, alone, is a nonbinding protein and no other SGBP might be involved in oligosaccharide or polysaccharide utilization that relies on the ability of the SusC/D-like complex to recognize and transport its cognate substrate.

## MATERIALS AND METHODS

### Heterologous protein expression and purification.

The gene fragment encoding the F5_SusD-like protein was PCR amplified from the metagenomic DNA of clone F5 (GenBank accession number HE717017), previously isolated from a metagenomic library derived from a human fecal sample (31). The PCR forward primer F5_SusD_Cloning_F (see Table S2 in the supplemental material) includes the NdeI restriction site, and the reverse primer F5_SusD_Cloning_R includes the XhoI site. The first 30 amino acids of F5_SusD-like corresponding to the lipoprotein signal peptide predicted by the SignalP 4.1 and LipoP 1.0 servers were not included in the PCR amplicon (51, 52). The gene product was cloned into the NdeI and XhoI restriction sites of the pET-28a(+) vector (Novagen, Darmstadt, Germany) preceding an N-terminal 6×His tag for affinity purification and transformed into E. coli DH5α (Invitrogen). The pET-28a(+) vector expressing F5_SusD-like was fully sequenced and transformed into E. coli BL21(DE3) for protein production. A single bacterial colony was inoculated into 5 mL of LB medium containing kanamycin (50 μg/mL) at 37°C. A preculture grown overnight was used to inoculate 200 mL of LB medium containing 50 μg/mL of kanamycin at an initial optical density at 600 nm (OD_600_) of 0.05. The culture was induced by the addition of 0.5 mM isopropyl β-d-1-thiogalactopyranoside (IPTG) in the mid-exponential phase (OD_600_ of ~0.6) and then incubated for 4 h at 37°C.

10.1128/msphere.00244-22.10 TABLE S2 List of PCR primers used in this study. Download Table S2, DOCX file, 0.01 MB. Copyright © 2022 Tauzin et al. 2022 Tauzin et al. https://creativecommons.org/licenses/by/4.0/ This content is distributed under the terms of the Creative Commons Attribution 4.0 International license.

For the purification of the recombinant F5_SusD-like protein, cells were harvested by centrifugation (15 min at 6,000 × *g*) and sonicated in Tris buffer (20 mM Tris-HCl, 300 mM NaCl [pH 8.0]). Bacterial debris was cleared by centrifugation at 11,000 × *g* for 30 min at 4°C, and the lysates were then passed through a column of 2 mL of Talon metal affinity resin (Clontech, USA). The F5_SusD-like protein was eluted using a gradient of 50 to 200 mM imidazole and then resuspended in Tris buffer (20 mM Tris-HCl, 150 mM NaCl [pH 7.5]) using an Amicon Ultra filter (Sigma-Aldrich) to eliminate the imidazole. Protein purity was confirmed via SDS-PAGE, and the concentration was determined by the absorbance at 280 nm using the Thermo Scientific Nanodrop 2000 instrument with an extinction coefficient of 153,350 M^−1^ cm^−1^. The F5_SusD-like protein was kept at 4°C for later analysis. The protein used for crystallization experiments was purified using an ÄKTAxpress system (GE Healthcare) with an affinity step (Talon crude, 1 mL; GE Healthcare) using the same loading buffer (20 mM Tris-HCl, 300 mM NaCl [pH 8.0]) and eluted with a 250 mM step of imidazole, followed by a gel filtration step (HiPrep 16/60 Sephacryl S-200 HR) in a solution containing 20 mM Tris-HCl and 0.15 M NaCl (pH 7.5). The fractions containing the monomeric F5_SusD-like protein were pooled, concentrated, and stored at 4°C.

### Glycosides.

The mixture of XOSs contains chains of DP2 to -7 (Wako Chemicals and IRO TAIHE). Simple xylans, with sparsely decorated structures, were purchased from Sigma for beechwood xylan, Biochemika Fluka for oat spelt xylan, and Megazyme for birchwood xylan and wheat arabinoxylan. Xylobiose, xylotriose, and 3^2^-α-l-arabinofuranosyl-xylobiose (AX2) were purchased from Megazyme. Barley β-glucan and xyloglucan from tamarind seed were also purchased from Megazyme. Laminarin from Laminaria digitata was purchased from Sigma.

### Isothermal titration calorimetry.

Isothermal titration calorimetry (ITC) analysis of glycan binding by F5_SusD-like was performed using the ITC200 calorimeter (Malvern), calibrated to 25°C. Proteins (40 μM) were prepared in buffer containing 20 mM HEPES–100 mM NaCl (pH 7.0), and oligosaccharides were prepared using the same buffer. The F5_SusD-like protein was placed into the sample cell, and the syringe was loaded with 2 to 10 mM XOS.

### Affinity gel electrophoresis.

The ability of F5_SusD-like to bind polysaccharides was assayed by affinity gel electrophoresis. Continuous native polyacrylamide gels were prepared, consisting of 10% (wt/vol) acrylamide in buffer containing 25 mM Tris and 250 mM glycine (pH 8.3). The substrates were added prior to polymerization at a final concentration of 0.5% (wt/vol). A total of 2.5 μg of purified F5_SusD-like was loaded onto the gels. Electrophoresis was carried out for 90 min on ice. Bovine serum albumin (BSA) was used as a noninteracting negative-control protein. The percentage of retention of F5_SusD-like in the gel containing glycans was calculated as (*D*_wog_ − *D*_wg_)/(*D*_wog_) × 100, where *D*_wog_ is equal to *D*_SusD-like_wog_/*D*_BSA_wog_ (the distance of migration of the lowest and most intense F5_SusD-like band normalized to the distance of migration of BSA in the gel without glycan) and *D*_wg_ is equal *D*_SusD-like_wg_/*D*_BSA_wg_ (the distance of migration of the lowest and most intense F5_SusD-like band normalized to the distance of migration of BSA in the gel with glycan).

### NMR spectroscopy.

All experiments were performed on a Bruker Avance II 800-MHz NMR spectrometer equipped with a QCPI cryogenically cooled probe head. Spectra were recorded at 298 K, and all samples were prepared in Tris buffer (20 mM Tris and 150 mM NaCl at pH 7.5). 1D proton spectra were acquired using Watergate water suppression (53), with 16 scans for XG_SusD-like (at 327 μM) and 256 scans for F5_SusD-like (at 54 μM). STD experiments were performed with saturation of the protein resonances at 0 ppm through a train of 5-ms Gaussian 180° pulses (34, 54). Typical experiments were run with 256 scans, 2,048 acquisition points, and a 5-s relaxation delay (including the 3-s presaturation train). STD experiments were run with 256 or 512 scans. Spectra were transformed after one level of zero filling and apodization with a π/3-shifted square sine bell.

### Crystallization and data collection.

The purified F5_SusD-like protein was concentrated to 20 mg/mL, and initial crystallization conditions were screened using a Mosquito robot (TPP Labtech) and JCSG I to IV commercial screens (Qiagen), from which one starting condition was identified (1.26 M sodium citrate, 0.09 M HEPES-HCl, 10% glycerol [pH 7.5]). After manual condition optimization, F5_SusD-like crystals were grown using the hanging-drop method by mixing 1 μL of the protein solution with 1 μL of the precipitant solution (1.7 M Na_3_-citrate, 10% glycerol, 0.1 M HEPES [pH 7.5]) and incubating the solution on a 24-well crystallization plate at 12°C. After harvesting, the crystals were soaked in cryoprotectant buffer (1.7 M Na_3_-citrate, 15% glycerol, 0.1 M HEPES [pH 7.5]), with or without the presence of 100 mM xylotriose, and flash-frozen in liquid N_2_. Data were collected at the XALOC beamline of the ALBA Synchrotron Light Source (Barcelona, Spain). A long-wavelength data set was also collected at a wavelength of 2.0 Å, using a special XALOC setup with continuous helium flow around the crystal to reduce absorption from the air (helium cone).

### Structure resolution and refinement.

Using the native data set only, the structure was solved using the Morda Web server (55), which identified a low-homology molecular replacement solution. This starting model was combined with the long-wavelength data set as implemented in the Phaser program (56) and used to calculate improved phases. This also enabled the correct positioning of all of the sulfur atoms in the sequence. The structure was determined via several rounds of modeling using the Arp/Warp Web server (57), alternated with manual rebuilding using Coot (58), and passed to Refmac for restrained refinement (59). The final model was validated with MolProbity (60) and WhatIF (61).

### Data availability.

The sequence of F5_SusD-like is available in the GenBank database under accession number CCG34975.1. The crystal structure of F5_SusD-like has been deposited in the Protein Data Bank under accession number 7NEK.
