# Supplementary figures and images for: Structural and Biochemical Characterization of a Nonbinding SusD-Like Protein Involved in Xylooligosaccharide Utilization by an Uncultured Human Gut Bacteroides Strain
Source: mSphere. 2022 Aug 31;7(5):e00244-22. doi: 10.1128/msphere.00244-22 (PMC9599597; doi:10.1128/msphere.00244-22)

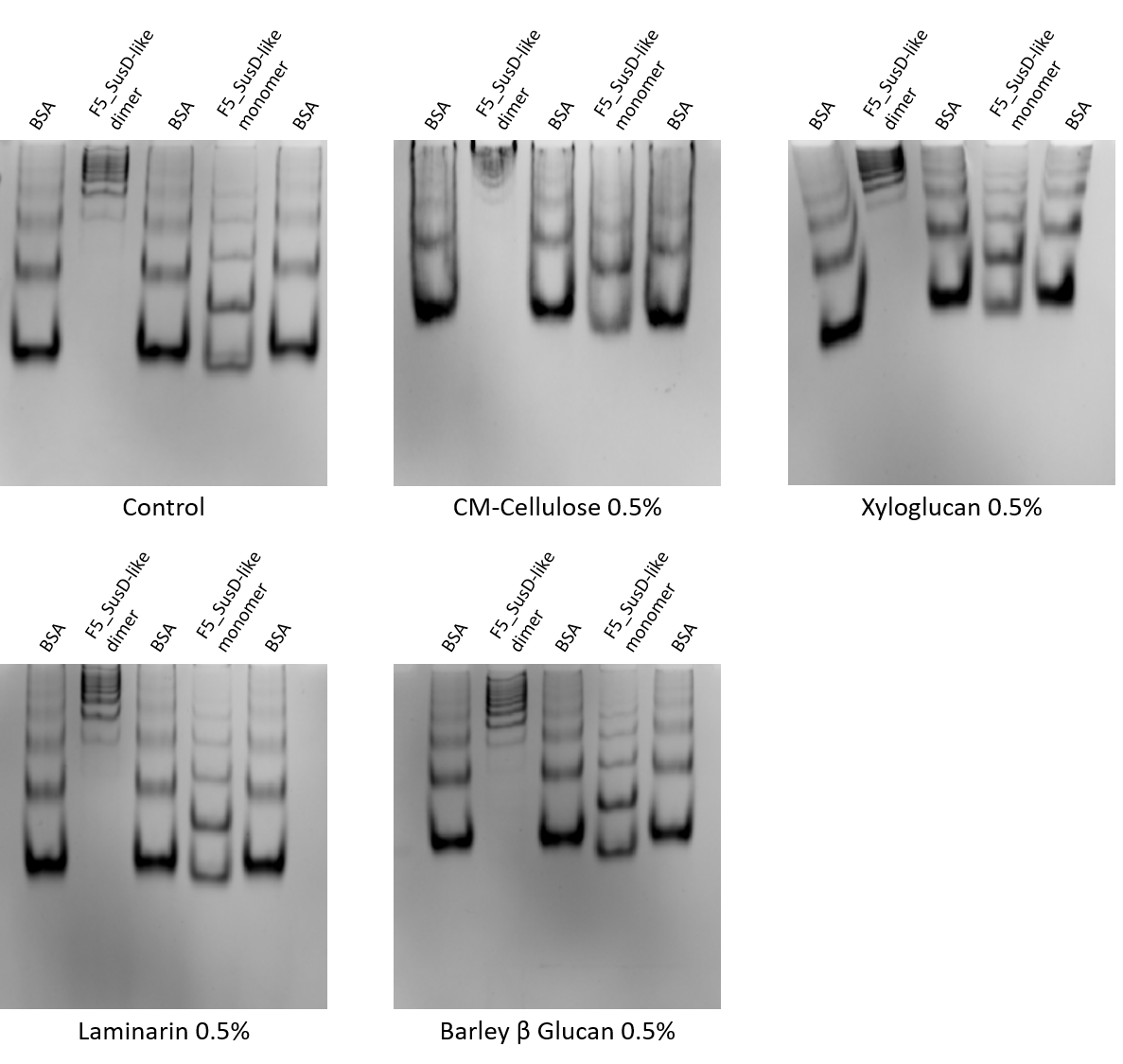

Supplement: FIG S1 [file msphere.00244-22-s0001.tif]

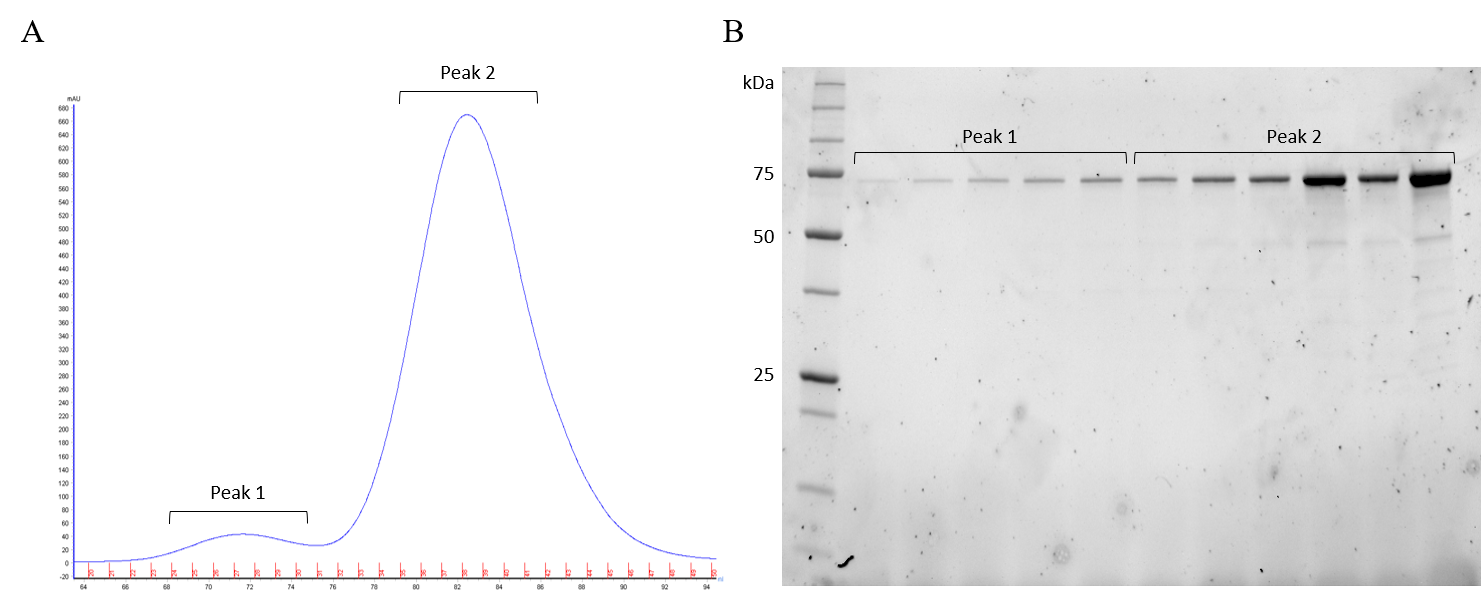

Supplement: FIG S2 [file msphere.00244-22-s0002.tif]

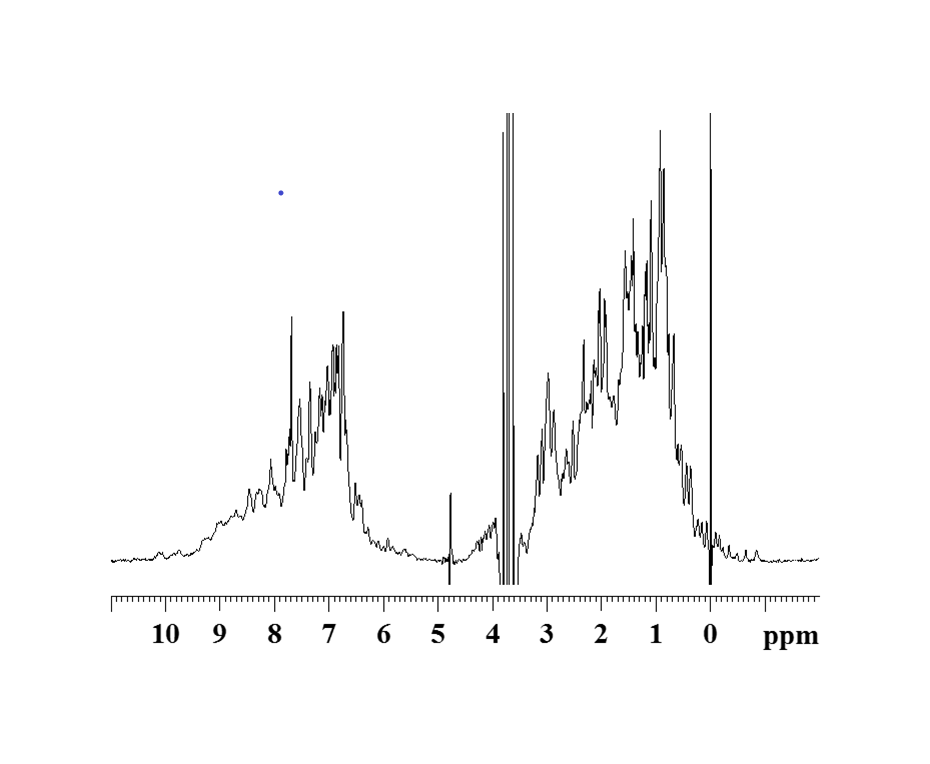

Supplement: FIG S3 [file msphere.00244-22-s0003.tiff]

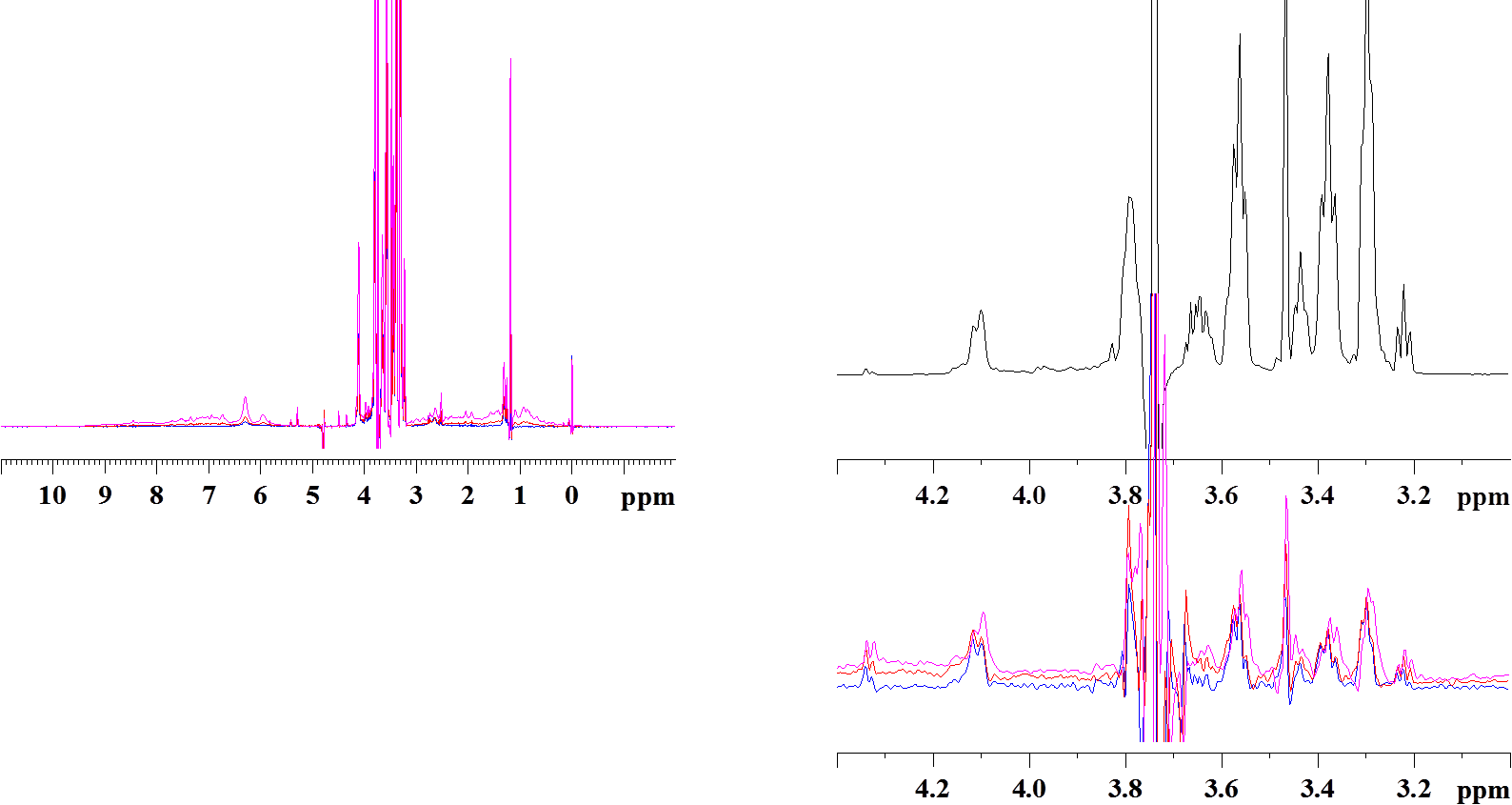

Supplement: FIG S4 [file msphere.00244-22-s0004.tif]

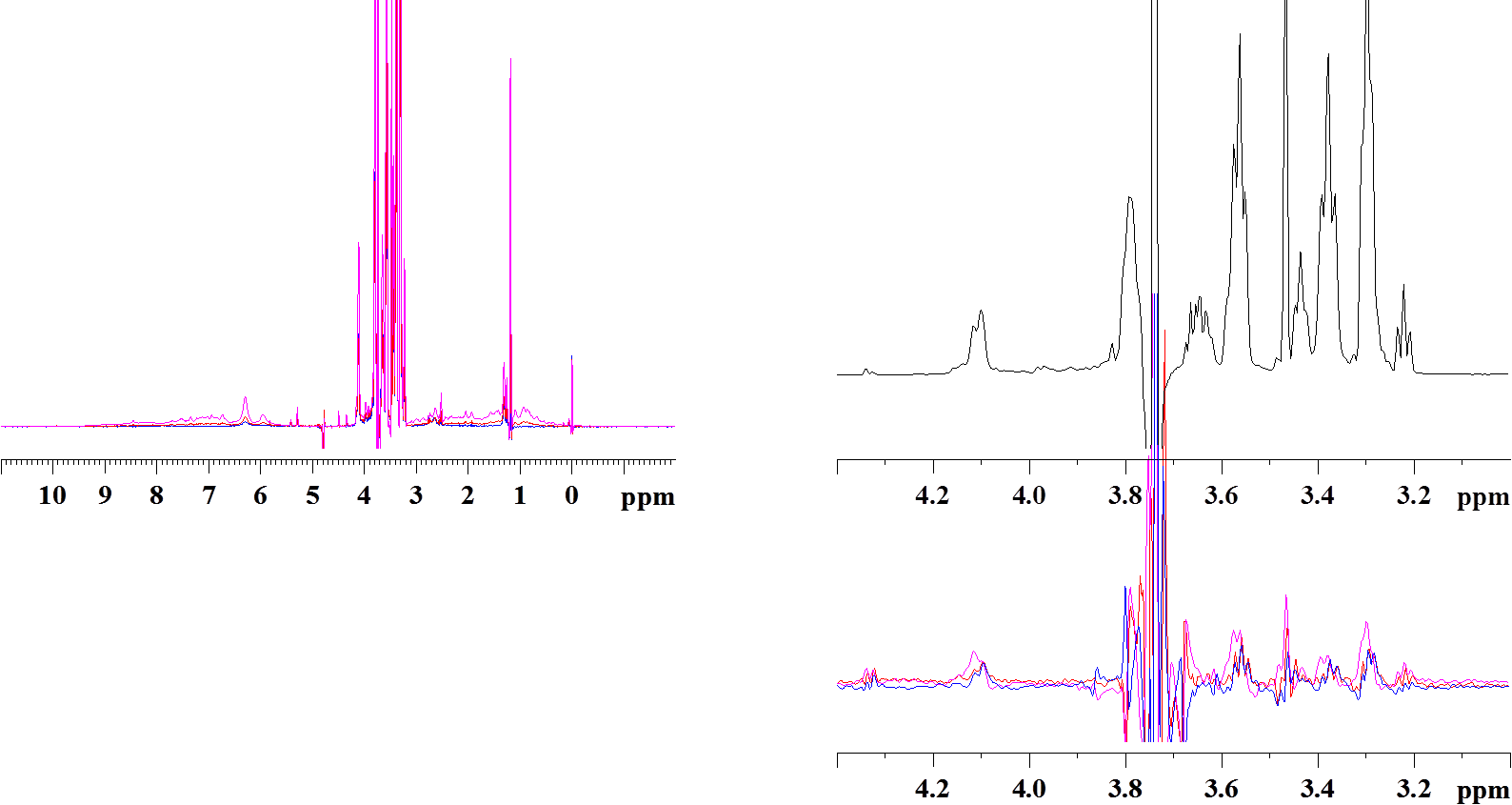

Supplement: FIG S5 [file msphere.00244-22-s0005.tif]

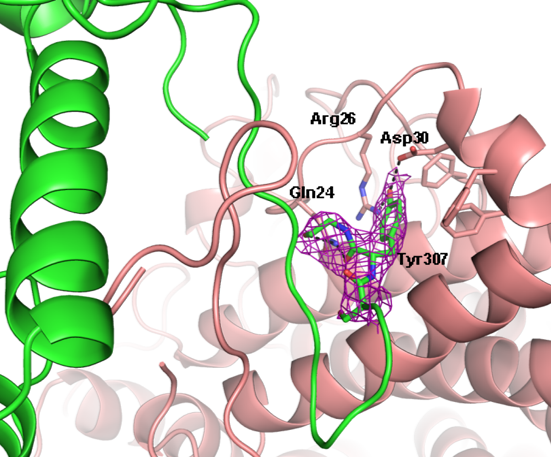

Supplement: FIG S6 [file msphere.00244-22-s0006.tif]

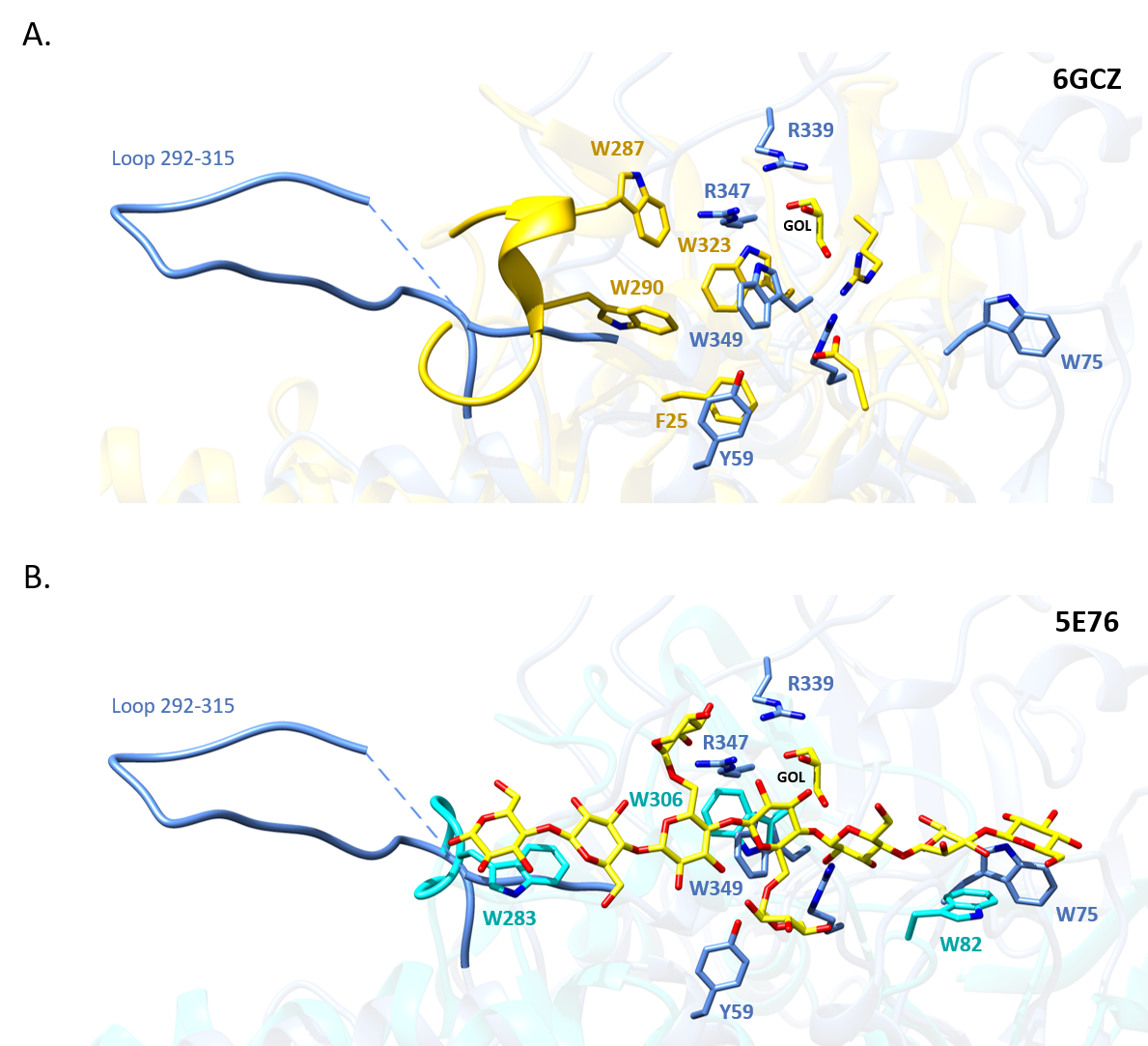

Supplement: FIG S7 [file msphere.00244-22-s0007.tif]

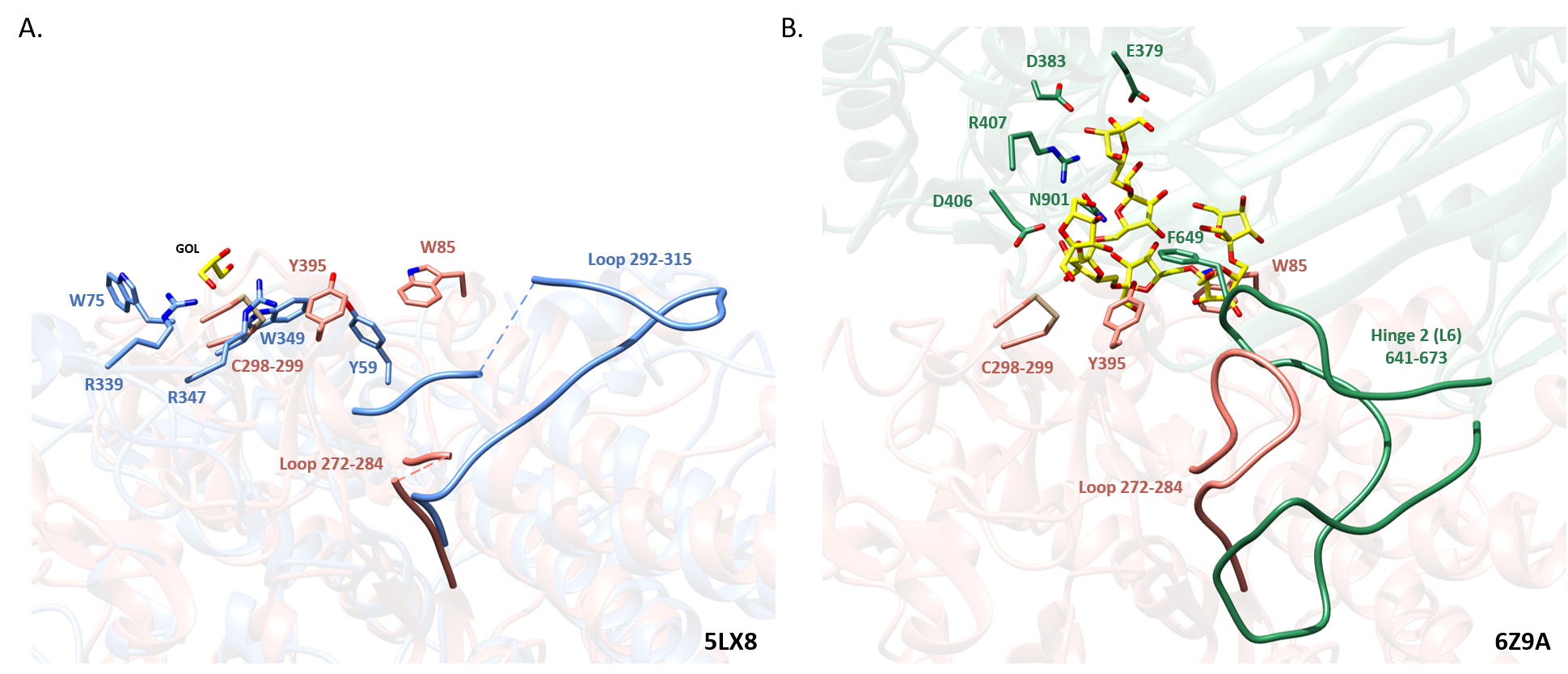

Supplement: FIG S8 [file msphere.00244-22-s0008.tif]
