# Supplementary material for: Structural and Biochemical Characterization of a Nonbinding SusD-Like Protein Involved in Xylooligosaccharide Utilization by an Uncultured Human Gut Bacteroides Strain
Source: mSphere. 2022 Aug 31;7(5):e00244-22. doi: 10.1128/msphere.00244-22 (PMC9599597; doi:10.1128/msphere.00244-22)
Supplement: TABLE S1 [file msphere.00244-22-s0009.docx]

| **Protein name** | **% identity with F5_SusD-like  (mature proteins)** | **Molecular weight (kDa) (mature proteins)** |
| --- | --- | --- |
| **F5_SusD-like** | 100 | 69.2 |
| **BACDOR_00535** | 99.68 | 69 |
| **BVU_0037** | 99.36 | 69.1 |
| **BACINT_01037^(1)^** | 26.01 | 62.4 |
| **HMPREF9447_02533^(1)^** | 25.34 | 65 |
| ***BACOVA_04392^(2)^** | 23.89 | 59.8 |
| **BACCELL_02148^(1)^** | 22.15 | 63.5 |
| **BACOVA_03429^(2)^** | 21.94 | 65.2 |
| **BXY_29260^(3)^** | 21.39 | 69.5 |
| **BXY_29280^(3)^** | 20.76 | 63.9 |
| ***BACOVA_03427^(2)^** | 19.43 | 69 |
