# Supplementary material for: Structural and Biochemical Characterization of a Nonbinding SusD-Like Protein Involved in Xylooligosaccharide Utilization by an Uncultured Human Gut Bacteroides Strain
Source: mSphere. 2022 Aug 31;7(5):e00244-22. doi: 10.1128/msphere.00244-22 (PMC9599597; doi:10.1128/msphere.00244-22)
Supplement: TABLE S2 [file msphere.00244-22-s0010.docx]

| **Name** | **Sequence 5’→3’** |
| --- | --- |
| F5_SusD_Cloning_F | CCGCGCGGCAGCCATATGGACGAGCAGCCGCGCAGCAGTTATG |
| F5_SusD_Cloning_R | GTGGTGGTGGTGCTCGAGTCAATCTCTGTATCCCGGATTCTG |
